# Intense Pulsed Light Therapy in the Treatment of Dry Eye Diseases: A Systematic Review and Meta-Analysis

**DOI:** 10.3390/jcm12083039

**Published:** 2023-04-21

**Authors:** Lilian Demolin, Majda Es-Safi, Muhammad Shahnawaz Soyfoo, Elie Motulsky

**Affiliations:** 1 Faculty of Medicine, Free University of Brussels, 1070 Brussels, Belgium; 2 Ophthalmology Department, Erasme Hospital, Hôpital Universitaire de Bruxelles, Faculty of Medicine, Free University of Brussels, 1070 Brussels, Belgium; 3 Rheumatology Department, Erasme Hospital, Hôpital Universitaire de Bruxelles, Faculty of Medicine, Free University of Brussels, 1070 Brussels, Belgium

**Keywords:** intense pulsed light, dry eye disease, meibomian gland dysfunction, tear break up time, non-invasive break-up time, ocular surface disease index, standard patient evaluation of eye dryness

## Abstract

Background: Intense pulsed light therapy (IPL) is a recently developed way of treating dry eye disease (DED). During the last decade, there was a multiplication of trials studying IPL efficacy. The goal of this review is to summarize the most important and significant results of these trials estimating effect sizes. Methods: The PubMed and sciencedirect databases were searched using a PICO model-based approach. Randomized controlled trials including at least 20 patients with DED and no other eye condition, with a control group and break-up time or symptom scores data available for extraction were included in this review. Statistical analysis evaluated the tear break-up time (TBUT), non-invasive break-up time (NIBUT), ocular surface disease index (OSDI), and standard patient evaluation of eye dryness (SPEED). Three comparisons were carried on for each outcome: longest follow-up values vs. baseline in the treatment group, longest follow-up values in the treatment group vs. control group, and changes from baseline in the treatment group vs. control group. A subgroup analysis was carried on. Results: Eleven randomized controlled trials, published between 2015 and 2021 were included in this systematic review with 759 patients in total. The longest follow-up values vs. baseline in the treatment group analyses were significantly in favor of IPL for all the parameters studied for instance: NIBUT (effect size (ES), 2.02; 95% confidence interval (CI), (1.43; 2.62)), TBUT (ES, 1.83; 95% CI, (0.96; 2.69)), OSDI (ES, −1.38; 95% CI, (−2.12; −0.64)) and SPEED (ES, −1.15; 95% CI, (−1.72; −0.57)). The longest follow-up values in the treatment group vs. control group analyses, and, the change from baseline in the treatment group vs. control group analyses, were both significantly in favor of IPL for NIBUT, TBUT, and SPEED but not for OSDI. Conclusions: IPL seems to have a positive effect on tear stability evaluated by the break-up times. However, the effect on DED symptoms is less clear. Some confounding factors such as the age and the IPL device used influence the results indicating that the ideal settings still need to be found and personalized for the patient.

## 1. Introduction

Dry eye disease (DED) is a very common issue as its prevalence ranges from 5 to 50% following the criteria and populations studied [1]. Although that frequent, it has only been recognized as a disease in 1995 and the latest definition was achieved in 2017 [2]. We are now aware of the multifactorial origin of the disease and the physio-pathological continuum existing between the different subtypes: aqueous deficient, evaporative, or mixed disease [2,3]. Some of the most important risk factors and etiologies of DED are systemic diseases like Sjögren syndrome, scleroderma, rosacea, or obstructive sleep apnea-hypopnea syndrome. Other risk factors are contact lens wearing, refractive surgery, demodex infestation, or smoking. Finally, adverse drug reactions, amongst others, antipsychotics, antidepressants, glaucoma medication, or anticholinergics [3,4,5,6,7,8] can also be a DED cause. The leading cause of evaporative dry eye disease remains the meibomian gland dysfunction (MGD) which is characterized by a vicious circle of inflammation, destruction of the meibomian glands, and dysfunction of the lipid layer of the tears. The composition of the tear film, therefore, becomes unbalanced and its stability is altered [9]. To target various points of that vicious circle, many different treatments of DED are available. Conventional therapy consists in educating the patient, controlling the risk factors, and teaching eyelids hygiene with warm compresses and artificial tears instillation. Then, depending on the causes of the DED, topical treatment like antibiotics, steroids, secretagogues or immunomodulators can be prescribed [10]. More recently, heat and light therapies have been developed. One of the very first devices available in clinics was the vectored thermal pulsation system known as Lipiflow^®^ which has shown good results in a recent meta-analysis [11]. Intense pulsed light therapy (IPL) was first used for dermatological conditions and in the cosmetic domain before being introduced in ophthalmology for DED treatment about 15 years ago [10]. IPL consists of flashes of light in a range of wavelengths from the visible (515 nm) to the infrared (1200 nm) that are delivered around the eyelids and absorbed by the tissues generating heat. Different intensities can be delivered depending on the Fitzpatrick skin classification. The eyes are protected from light by opaque goggles. This can be repeated in several sessions separated by a few weeks according to different schedules [12]. The mechanisms by which IPL has an effect on the relief of DED symptoms are various and still partly unclear [13]. The leading hypothesis is the eradication of demodex, the thrombosis of abnormal vessels, the fluidification of meibum, and photobiomodulation [13,14,15]. In the last decade, studies evaluating the efficacy and safety of IPL therapy in DED appeared. The goal of this meta-analysis review is to determine the efficacy of IPL in DED.

## 2. Materials and Methods

This literature review and meta-analysis were realized according to the Preferred Reporting Items for Systematic Reviews and Meta-Analysis (PRISMA) [16].

### 2.1. Inclusion Criteria

The following inclusion criteria were defined:

1. Paper published either in English or French.
2. Trials including more than 20 patients.
3. Adult patients diagnosed with DED or MGD and no other conditions that could affect the different assessments such as acute inflammation, contact lens wearing, previous IPL treatment, or other local eyelid treatment.
4. Trials that compared IPL therapy ± meibomian gland expression (MGX) with sham treatment, eyelid hygiene, MGX alone, or no treatment.
5. Any of the following data available for extraction: Tear break-up time (TBUT) [17], Non-invasive break-up time (NIBUT) [18], Ocular surface disease index (OSDI) [19,20], SPEED [21,22].

### 2.2. Search Strategy

The PICO model [23] was used to define the following research question: “Among adults with dry eye syndromes, an intense pulsed light therapy improve the tear break-up time and the symptoms scores compared to a placebo, sham treatment or lid hygiene?”. Then, the Pubmed and ScienceDirect databases were searched using keywords such as “Dry eye syndrome”, “Keratoconjunctivitis sicca”, “Meibomian gland dysfunction”, “Tear break-up time”, “OSDI”, “SPEED”, “Intense pulsed light” and “IPL”. The Boolean operators “AND” and “OR” were used. Articles published between 2011 and 2022 were imported into Endnote X9.

### 2.3. Selection Process and Data Extraction

Using the PICO model, we looked in the Pubmed and ScienceDirect database, resulting in a large number of studies. The selection process to retain the articles was performed in three different phases: duplicates removal, title, and abstract screening, and finally full-text review. Duplicates were screened and removed by the Endnote automation tool. A manual double-check for duplicates was also realized. After the removal of the duplicates, one reviewer (DL) screened each record. Papers failing to satisfy the inclusion criteria via title and abstract were eliminated. Other papers were screened by reading the full text. Data from eligible records were extracted and imported to Excel and SPSS for statistical analysis. Break-up times and symptom scores outcomes of the baseline, longest follow-up, and change from baseline in the form of mean ± standard deviation (SD) were collected from article data. If necessary, the SD was computed from a 95% confidence interval or standard error of the mean. Results in the form of the median and interquartile range were not extracted. Other variables such as sample size, patient characteristics, control group intervention, power used, number of treatments, and their schedule and length of the longest follow-up were extracted (Supplementary Materials Table S1).

### 2.4. Risk of Bias Assessment

The Cochrane risk of bias tool for randomized trials (ROB 2 Tool) was used to assess the risk of bias in selected studies [24]. Studies were classified as “low risk”, “some concerns” or “high risk” for five different biases: randomization process, deviation from the intended interventions, missing outcomes data, measurement of the outcome, and selection of reported results.

### 2.5. Statistical Analysis

Statistical analysis was realized with the SPSS software. For each outcome, Cohen’s d was calculated between the treatment and control group for the change from baseline and the longest follow-up outcomes. Inside the treatment group, Cohen’s d was calculated between the longest follow-up and baseline outcomes. An inverse-variance weighting was then applied to compute a weighted mean between Cohen’s ds. Symptom scores outcomes of studies using one eye in the treatment group and the fellow eye as control was not included in the analysis as both OSDI and SPEED cannot evaluate eyes individually. Change from baseline outcomes that were not specified in the paper was calculated using the mean difference between the longest follow-up and baseline outcomes. Forest plots for each comparison were then generated by the SPSS software. A random effect model was used, and the I-squared test was used to assess the heterogeneity of the results. To investigate the origin of heterogeneity, a sub-group analysis was carried on. Different sets of subgroups were used. The first was based on the addition of meibomian gland expression to the IPL treatment or not. The second was based on the different IPL devices used in the studies. The third set was based on the mean age of the subjects. The fourth set was based on the control group consisting of a sham treatment or no treatment. Finally, the last set was based on gender. A chi-square test was used to determine if the subgroups showed a statistical difference between their results. To further analyze the amount of heterogeneity brought by each study, a sensitivity analysis was carried on by removing each of them one by one and observing the variation of heterogeneity. Results were considered robust when the changes of heterogeneity were under 10% withdrawing studies one by one. The risk of reporting bias was assessed by generating funnel plots and examining their symmetry.

## 3. Results

### 3.1. Study Selection

**Figure 1:**
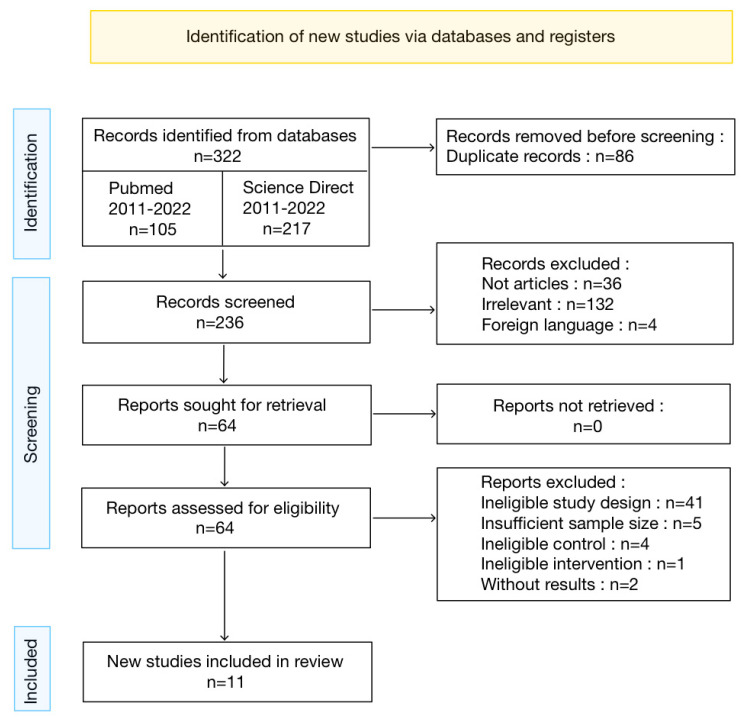
PRISMA flowchart diagram.

The database search using the previously defined keyword identified 322 records from which 86 duplicates were removed (Figure 1). A first screening based on the titles helped to eliminate 172 records that were not articles, were irrelevant, or were published in another language than French and English. A total of 64 records were screened through abstract screening and later full-text review. From these 64 records, 11 studies were assessed as eligible and included in this meta-analysis, the 53 others were excluded. Our selection method is presented in detail in the PRISMA Flowchart Diagram (Figure 1). Our very restrictive selection criteria excluded some articles such as an article that was evaluating the efficacy of IPL followed by meibomian gland dysfunction compared to IPL alone [25], or another study that analyzed the level of cytokines in tears and made a correlation with the other, more standard, outcomes [26]. Although it is an interesting perspective, since it was the only article analyzing this outcome, it was not possible to include it in the present meta-analysis. Finally, two articles were comparing other new types of treatment with the IPL. The first compared IPL with meibomian gland probing and with the combination of both treatments [27]. The other [28] compared the near-infrared light treatment with the IPL.

### 3.2. Study Characteristics

All eleven studies included in the meta-analysis were published in English. Six of them [29,30,31,32,33,34] were realized in China. Two [12,35] are from New Zealand. The others come from diverse countries: Japan [36], Thailand [37], and Iran [38]. They were all published between 2015 [12] and 2021 [31,32,38]. One study [29] concerns post-LASIK dry-eye syndrome while all the others concern meibomian gland dysfunction. No trial concerning rosacea, Sjögren’s syndrome, or other dry-eye syndromes fitting the eligibility criteria were found. The number of patients included in the studies ranges from 28 [12] to 120 [31] and the total number of patients included in this meta-analysis is 759. Most of the studies have a mean age of patients above 40 years [12,30,31,33,34,35,36,37,38]. Nevertheless, other studies have been performed on younger patients like Pazo et al. [29] where the mean age was 30.5 ± 5.2 in the treatment group, or like Song et al. [32] who had a mean age of 28.2 ± 3.6 in the treatment group. This difference in population age could lead to differences in the results. Subgroup analysis will therefore be carried on. All the studies have a sex ratio more feminine. Two different IPL devices were used in the collected studies: E > Eye from E-SWIN for four of the studies [12,35,37,38] and the M22 from Lumenis for the others [29,30,31,32,33,34,36]. The power used depends on the Fitzpatrick skin type and ranges from 9 to 17 J/cm^2^. Most of the studies [12,31,32,33,34,37,38] had a 3-IPL session schedule with several weeks in between but Gao et al. [30] had only one session and Arita et al. [36] had a total of eight sessions with 3-week intervals. The treatment groups underwent IPL treatment sessions with the addition of meibomian gland expression in three studies [31,33,36]. Xue et al. [35] have two treatment groups. One with four flashes of light delivered per session and per eye, and another group with five flashes of light delivered per session and per eye. Both groups were included in the meta-analysis. Two studies [12,33] have a paired-eye design with one eye receiving the IPL treatment and the other eye receiving a sham treatment. The other studies have a parallel group design with the control group receiving a sham treatment [32,35,37], meibomian gland expression [31,36], warm compresses or other eyelid hygiene measures [31,34,38], antibiotics (Tobramycine/dexamethasone) [30], or no treatment [29]. Finally, the longest follow-up outcomes were collected in a range going from one month [29,30] to 32 weeks [36]. Studies characteristics are summarized in Table 1.

### 3.3. Risk of Bias in Included Studies

**Figure 2:**
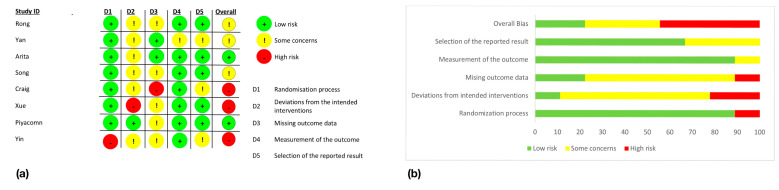
Risk of bias assessment: (**a**) Evaluation of individual studies; (**b**) Overall amount of each bias [12,29,30,31,32,33,34,35,36,37,38].

The risk of bias in included studies, assessed by the ROB2 assessment tool [24] is presented in Figure 2a,b. For the five following biases: randomization process, deviation from the intended intervention, missing outcome data, measurement of the outcome, and selection of the reported results, studies are assessed as “low risk”, “some concerns” or “high risk”. The randomization process was assessed as “low risk” for five studies [12,30,33,35,38]. Two studies [31,37] had a randomization process working with algorithms such as stratified block randomization [37] and interactive web response system [31] that could lead to randomization process bias. They, however, did not show any baseline differences and were therefore assessed as “some concerns” for randomization process bias. Two studies [32,36] mentioned being randomized but did not explain the randomization process. They did not suffer from baseline differences either and were also classified as “some concerns” for randomization process bias. Finally, two studies [29,34] did not mention being randomized and baseline differences in the number of subjects or the outcomes could indicate bias. They were classified as “high risk” for randomization process bias. Concerning the deviation from the intended intervention bias, all the studies had the physician delivering the intervention aware of the group of each patient. They must know whether they give a true IPL session or not. Five studies [12,32,33,35,37] were assessed as “low risk” for this bias as their patients received a sham treatment and were not aware of their intervention group. The six other studies [29,30,31,34,36,38] did not use any sham treatment and their patients were therefore aware of their intervention group. These studies were classified as “some concerns” for this bias. For the next bias, missing outcome data, nine of the studies [12,29,30,31,33,35,36,37,38] were assessed as “low risk” due to their less than 7% missing outcomes. Two studies [32,34] were classified as “high risk” for missing outcomes bias: Song et al. [32] had 18% of missing outcomes and did not give any explanation of the reason why patients dropped out of the study, Yin et al. [34] did not give any information about missing outcomes data. For the measurement of the outcomes, 7 out of the 11 studies [12,32,33,34,35,37,38] mentioned the blindness of the assessor and were ranked as “low risk” for measurement of the outcome bias. The four other studies [29,30,31,36] did not give information on whether the assessor was blind but no strong proof that the assessment of the outcomes was influenced by the knowledge of the intervention received was found. They were assessed with “some concerns” for this bias. Figure 2a shows the evaluation of each bias for each study and the computed overall bias. Figure 2b shows, for each bias, the percentage of studies evaluated as low risk, some concerns, or high risk.

### 3.4. Syntheses Results

**Figure 3:**
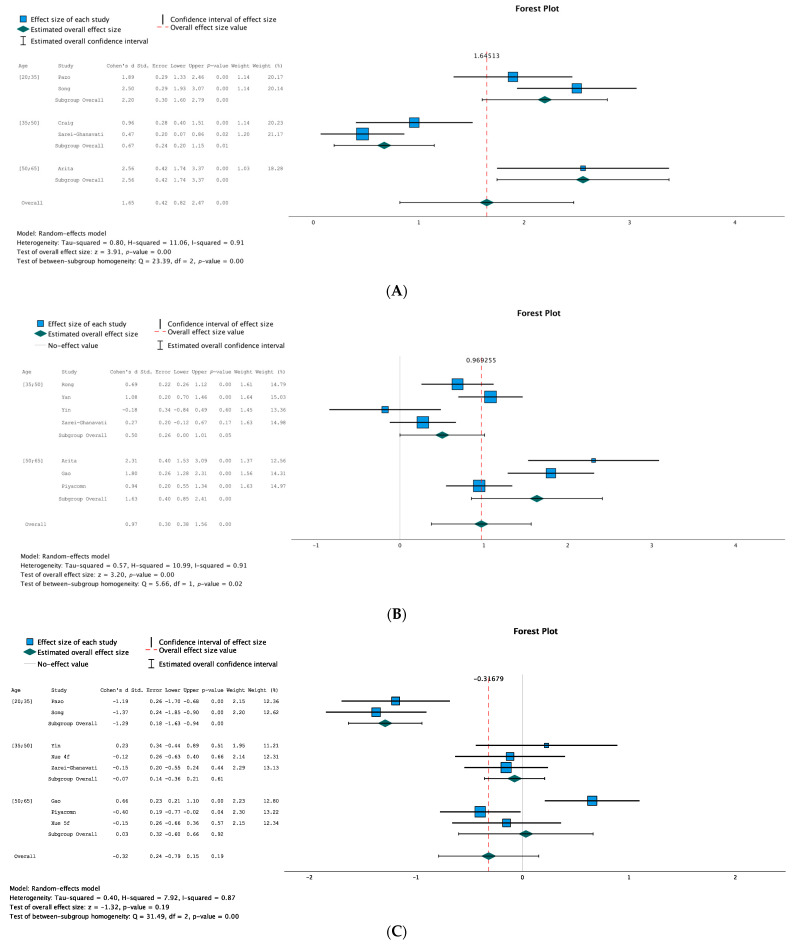

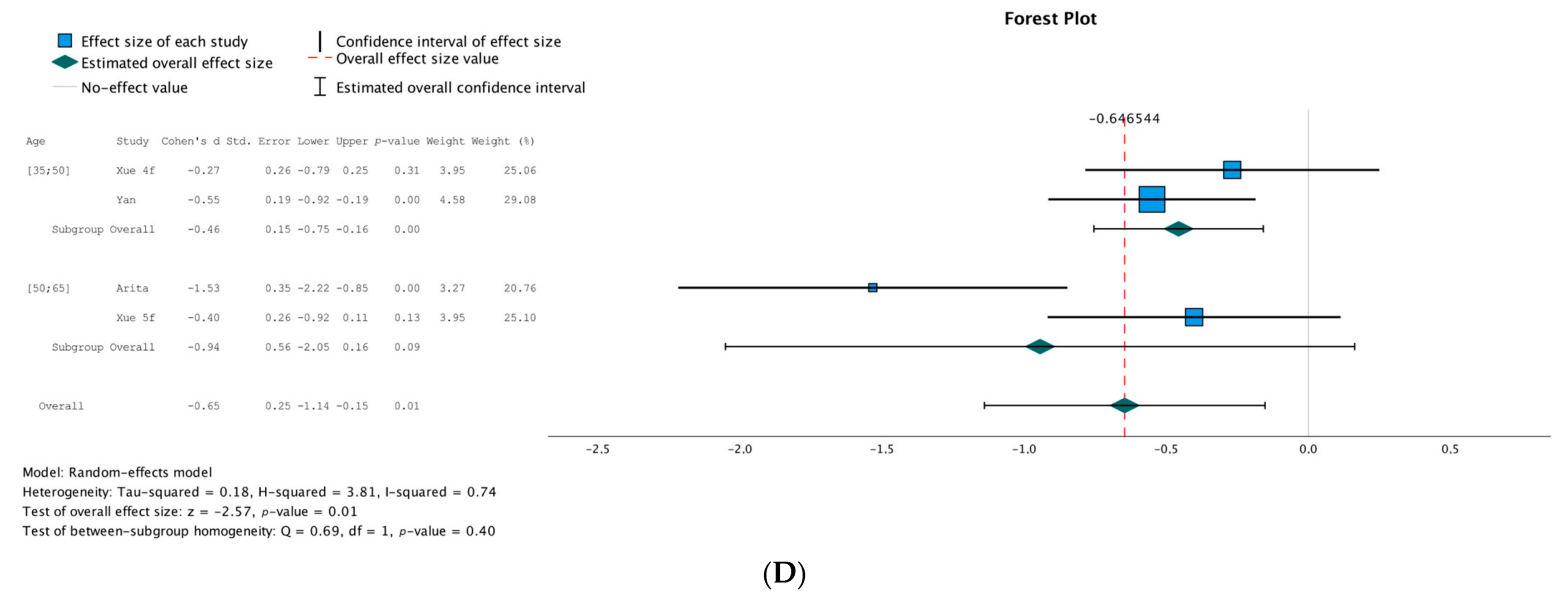
Legend: std = standard, 4f = 4 flashes, 5f = 5 flashes. (**A**). NIBUT: changes from baseline between groups, forest plot. (**B**). TBUT: changes from baseline between groups, forest plot. (**C**). OSDI: changes from baseline between groups, forest plot. (**D**). SPEED: changes from baseline between groups, forest plot [12,29,30,31,32,33,34,35,36,37,38].

This meta-analysis focused on the random-effects model and inverse variance weighted analysis. For each data collected (TBUT, NIIBUT, OSDI, SPEED), three continuous meta-analyses were performed. The first compared the post-treatment outcomes to the baseline outcomes in the treatment groups. The second compared the longest follow-up outcomes of the treatment groups to the control groups. The third compared the change from baseline in the treatment group to the control group. To lower the intervariance studies, the effect size was calculated as the standardized mean difference or Cohen’s d. The standard error of the mean, 95% confidence intervals, and *p*-value will also be reported. Finally, the heterogeneity will be evaluated by the I^2^ test value. Break-up times are meant to increase with successful treatment. In the treatment groups, we should therefore observe a positive change from the baseline for the BUT values. Comparing the treatment and control groups, longer break-up times post-treatment and bigger increases from baseline in the treatment groups should be found. NIBUT outcomes were reported in five studies [12,29,32,36,38]. The three NIBUT meta-analyses show significant differences in favor of the treatment group. The overall effect size for the difference between the longest follow-up and baseline in the treatment group is 2.02 standard deviation (SD); 95% CI, (1.43; 2.62), SE, 0.30; *p* < 0.01 and I^2^, 0.82. For the difference in longest follow-up NIBUT between the groups, the effect size is 1.66; 95% CI, (0.71; 2.60); SE, 0.48; *p* < 0.01; I^2^, 0.93. For the difference in changes from baseline between the groups, the effect size is 1.65, 95% CI, (0.82; 2.47); SE, 0.42; *p* < 0.01; I^2^, 0.91. TBUT outcomes were reported in seven studies (30,31,33,34,36–38). The TBUT meta-analysis also shows three significant differences in favor of IPL therapy. The difference between baseline and post-treatment values in the treatment groups has an effect size of 1.83; 95% CI, [0.96; 2.69]; SE, 0.44; *p* < 0.01; I^2^, 0.95. The difference between the groups for the longest follow-up TBUT has an effect size of 1.19; 95% CI, (10.34; 2.04); SE, 0.43; *p* = 0.01; I^2^, 0.95. The difference between the groups for changes from baseline has an effect size of 0.97; 95% CI, (0.38; 1.56); SE, 0.30; *p* < 0.01; I^2^, 0.91. Symptoms scores are supposed to decrease with successful treatment. In the treatment group, we should therefore observe a diminution of OSDI and SPEED scores from baseline. Comparing the treatment and control groups, lower scores post-treatment and bigger decreases from baseline in the treatment group should be found. OSDI outcomes were reported by seven studies [29,30,32,34,35,37,38]. The OSDI meta-analyses are less conclusive than the break-up times meta-analyses as only the difference between baseline and longest follow-up in the treatment group is significant, ES, −1.38; 95% CI, (−2.12; −0.64); SE, 0.38; *p* < 0.01; I2, 0.94. The difference in post-treatment OSDI between the groups, ES, −0.56; 95% CI, (−1.56; 0.04); SE, 0.30; *p* = 0.07; I^2^, 0.92 and the difference in changes from baseline between the groups, ES, −0.32; 95% CI, (−0.79; 0.15); SE, 0.24; *p* = 0.19; I^2^, 0.87 are both statistically non-significant. The last parameter collected in three studies is the SPEED outcome [31,35,36]. The SPEED meta-analyses show a significant difference in favor of the IPL treatment. In the treatment group, the difference between baseline and post-treatment is −1.15; 95% CI, [−1.72; −0.57]; SE, 0.29; *p* < 0.01; I^2^, 0.79. The difference between groups for longest follow-up values is −0.52; 95% CI, [−0.76; −0.27]; SE, 0.12; *p* < 0.01; I^2^, 0.03 and for changes from baseline is −0.65; 95% CI [−1.14; −0.15]; SE, 0.25; *p* = 0.01; I^2^, 0.74. The overall effect size of changes from baseline between the treatment and control groups are depicted in Figure 3A–D. In Figure 3A, we can observe that the confidence interval of effect size is positive and does not comprise 0. The same observation can be performed in Figure 3B. IPL has therefore a statistically significant effect in increasing the NIBUT and TBUT. Figure 3C presents the changes from baseline analyses for OSDI and shows that the confidence interval of overall effect size comprises 0 and that the results are not statistically significant. Finally, for the SPEED outcome, Figure 3D shows a negative confidence interval of overall side effect that does not comprise 0. We can conclude that IPL treatment significantly reduces the SPEED score.

### 3.5. Heterogeneity Analysis

According to the Cochrane handbook for a systematic review of intervention [39], all the NIBUT, TBUT, and OSDI meta-analyses were classified as having a considerable heterogeneity (I^2^ between 75% and 100%). The SPEED meta-analysis comparing the longest follow-up results between groups had a very low heterogeneity which is classified as “might not be important” (I^2^ = 0.03) whereas the one comparing changes from baseline between groups may have substantial heterogeneity (I^2^ = 0.74). To analyze the potential origins of this heterogeneity, subgroups analysis was undertaken on the changes from baseline between groups comparisons. The different sets of subgroups were the IPL device used (Lumenis or E-SWIN), the age (between 20 and 35, between 35 and 50, and between 50 and 65 years old), the sex ratio (more or less than one-third of males in the treatment group), the control group intervention (usage of sham treatment or not) and the addition or not of MGX after the treatment. These subgroups were chosen due to their potential influence on the results. A chi-square test was used to determine if the subgroups showed a statistical difference between their results. A *p*-value of 0.1 was used as a cut-off for statistical significance due to the poor number of studies and the low power of the test. No statistically significant differences were found in the different sets of subgroups for the SPEED meta-analyses. This could be explained by the fact that this comparison is the one with the least number of studies (including only three studies and four treatment groups) and the one with the lowest I^2^ test (0.74). For the NIBUT outcomes, a statistically significant difference was found between the subgroups based on the device used (Q = 23.42, *p* < 0.01), with the Lumenis group having a higher effect size. The addition or not of MGX also lead to a statistically significant difference between the subgroups (Q = 3.24, *p* = 0.07) although only one study was included in the MGX + group [37]. The age-based subgroup analyses are presented in Figure 3A–D. Age-based subgroups were significant for the NIBUT (Q = 23.39, *p* < 0.01), TBUT (Q = 5.66, *p* = 0.02), and OSDI (Q = 31.49, *p* < 0.01) outcomes with bigger effect sizes in younger groups. Sensitivity analysis was then undertaken to identify the impact of each individual study on the heterogeneity results in the changes from baseline between groups comparisons. The analyses were considered robust when no substantial change (less than 10%) in heterogeneity happened. Zarei-Ghanavati et al. [38] was identified as the study bringing the most heterogeneity in the NIBUT meta-analyses. Removing it makes the I^2^ test drop from 0.91 to 0.83. Arita et al. [36] was identified as the study bringing the most heterogeneity in the TBUT meta-analyses making the I^2^ test drops from 0.91 to 0.88 when removed. Heterogeneity remaining considerable in both cases, the results of the NIBUT and TBUT analyses were assessed as robust. The study bringing the most heterogeneity in OSDI analysis was Gao et al. [30], the only study having a change from baseline favoring the control group. I^2^ dropped from 0.87 to 0.83 when this study was removed. Results of the OSDI analysis were therefore also assessed as robust. Finally, the SPEED analysis was assessed as having low robustness due to a drop in the I^2^ test from 0.74 to 0.00 when removing Arita et al. [36] from the analysis.

### 3.6. Reporting Biases Assessment

**Figure 4:**
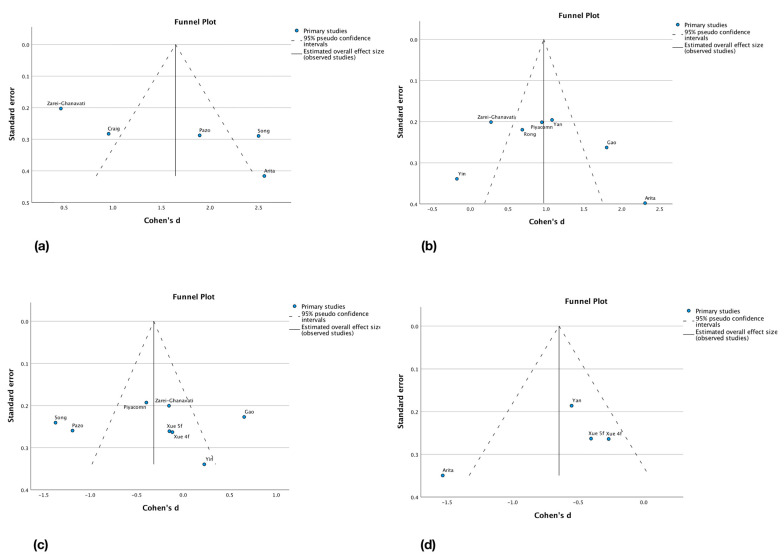
Reporting bias assessment, change from baseline between groups, funnel plots. (**a**) NIBUT; (**b**) TBUT; (**c**) OSDI; (**d**) SPEED [12,29,30,31,32,33,34,35,36,37,38].

Reporting biases were assessed by the interpretation of funnel plots realized from the changes from baseline between groups meta-analysis. However, having strict inclusion criteria and a low number of studies included in the meta-analyses, this interpretation is complicated and the symmetry of the plots is difficult to evaluate. This could be explained by the low amount of patients in included studies and by the high heterogeneity of groups and study designs. Globally, less symmetry can be found in the symptom scores outcomes plots. Funnel plots are presented in Figure 4a–d. Possible reporting biases sources are language bias as only articles in English and French were eligible, location bias, and publication bias.

## 4. Discussion

IPL therapy seems to have a good effect on the break-up times. Being measured with fluorescein or with non-invasive methods, we could observe a positive impact of the treatment. The NIBUT especially shows a strong effect size of 1.65 standard deviations taking into account that no more than five studies were included [12,29,32,36,38]. Obviously, larger studies are needed to evaluate the true impact on tear stability. Concerning the symptom scores, the impact of IPL therapy seems less conclusive. The OSDI score was the outcome with the highest number of results included in the analyses [29,30,32,34,35,37,38] and yet failed to reach a statistical difference between the groups. Analyses of the SPEED score outcome had the lowest statistically significant effect size and the lowest number of results included [31,35,36]. Their reliability is therefore impacted, and caution should be taken while interpreting the results. Symptoms are left to the appreciation of the patient, some being highly symptomatic, some being less, and some being totally asymptomatic for the same severity of the disease. This subjectivity may also lead to a stronger placebo effect [40]. When analyzing the treatment groups alone, the effect sizes are way bigger in NIBUT, TBUT, OSDI, and SPEED outcomes. The amount of the placebo effect is therefore hard to estimate. Further randomized trials, controlled by a sham treatment, and with a bigger sample size would help to address this issue. Subgroup analysis highlighted the fact that some factors could influence the treatment efficacy. The age of the patient may be one and large-scale studies could investigate that parameter for an adaptation of the treatment according to patient age. The difference found in the results of NIBUT following the device used could indicate that ideal settings still are to be found. The fluence, power, intensity, or number of flashes are different in all the studies and could therefore influence the results leading to a different efficacy of IPL devices. Other factors not analyzed here could also influence the results. The severity of the disease could be one, as MGD is characterized by a vicious circle that makes itself more severe over time [9]. The reaction of the glands to IPL therapy sessions may vary following the degree of inflammatory infiltration, atrophy, or drop-out. Meibographic data was insufficient to carry-on subgroup analysis based on the severity of the disease and, therefore, further studies analyzing the effect of IPL in correlation with meibographic findings are needed. New tools for the evaluation of the disease could also be implied in future studies. Artificial intelligence has seen a huge development this last decade and could soon be used for the quantification of meibomian glands. This quantification could be used to classify the patient according to the severity and prognosis of the DED. Treatment schemes and settings could then be chosen according to that classification [41]. Tear metabolomics is another emerging technology that could, via the identification of distinct metabolites or specific metabolic patterns, be used in the future for the classification of DED [42]. Etiologies leading to dry eye diseases are equally an important factor to consider. They all lead to different entry points in the vicious circle [9] and may react differently to the IPL treatment. Data was also insufficient to carry-on etiological subgroup analysis. It should be considered in further studies. The correlation with the usage of medication altering the quality or quantity of tears such as isotretinoin, glaucoma medication, and antidepressant [4,5,43], is another area that should further be explored. The present meta-analyses focused on four outcomes’ data: NIBUT, TBUT, OSDI, and SPEED. The little amount of data concerning other outcomes make them difficult to compare in a meta-analysis for now nevertheless further studies might evaluate these outcomes allowing a broader analysis. Important fields such as the impact on inflammatory cytokines [26], microbiological flora of the tears and lids [35], tear film interferometry [32] or once again meibographic findings [44] are pertinent parameters that should be included in future analysis. IPL therapy is slowly finding its place in the constantly evolving domain of evaporative dry eye disease. Lid hygiene measures are still the most common measures for the treatment of evaporative dry eye although they are now accompanied by complementary technologies and techniques [45]. A very recent systematic review [11] carried out a meta-analysis to evaluate the efficacy of vectored thermal pulsation system, finding encouraging results. While this system is generally considered a one-time procedure and reserved for mild to moderate diseases [11], IPL can be repeated and administered to moderate and severe cases. Chung et al. [46] even showed in a recent study that both technologies can be combined with good efficacy for refractory cases. Future trials should evaluate further this possibility of combination and its indication. Comparison of both treatments’ efficacy should also be a subject of further studies. Near-infrared light is another new technology that may have a positive impact on the dry eye even though this impact seems to be lesser than with IPL [27]. Another interesting treatment approach yet invasive, is a meibomian gland probing that showed good results in combination with IPL for the relief of dry eye symptoms [28].

## 5. Conclusions

Based on the results of this systematic review, IPL seems to increase the tear film stability as the break-up times are longer. The age of the subject also seems to have an impact on the efficacy of the treatment and the potential difference between IPL devices used may indicate that adequate settings still need to be refined and personalized for each patient. Further large-scale, randomized, sham-controlled trials are therefore needed to prove the efficacy of IPL on dry eye symptom relief. As scientific evidence around IPL grows in the future, its place in the treatment of evaporative dry eye and MGD should get clearer.

## Figures and Tables

**Table 1:** Characteristics of the studies included in the meta-analyses.

First Author	Country	Year	Number of Patients	Age (Years)	Sex Ratio M/F	Instrument Model	Power (J/cm^2^)	Frequency and Number of Sessions	TG	CG	Longest Follow-Up
Arita [36]	Japan	2019	42	TG: 61.0 ± 18.0 CG: 61.9 ± 12.2	TG: 9/13 CG: 8/12	A	11–14	3w 8 sessions	IPL + MGX	MGX only	32w
Craig [12]	New-Zealand	2015	28 (56 eyes)	45 ± 15	8/20	B	9–13	D0-D15-D45 3 sessions	IPL only	Sham (2nd eye)	45d
Gao [30]	China	2019	82	TG: 54.4 ± 16.2 CG: 55.2 ± 16.7	TG: 10/31 CG: 11/30	A	12–14	1 session	IPL only	AB	1m
Pazo [29]	China	2020	36	TG: 30.5 ± 5.2 CG: 31.0 ± 4.3	TG: 9/12 CG: 7/8	A	11–14	2w 2 sessions	IPL only	No trt	28d
Piyacomn [37]	Thailand	2020	114	TG: 59.0 ± 12.7 CG: 59.5 ± 11.4	TG: 10/47 CG: 5/52	B	9–13	D0-D15-D45 3 sessions	IPL only	Sham	6m
Rong [33]	China	2018	44 (88 eyes)	46.3 ± 16.9	12/32	A	14–16	4w 3 sessions	IPL + MGX	Sham + MGX (2nd eye)	3m
Song [32]	China	2022	71	TG: 28.2 ± 3.6 CG: 28.1 ± 3.7	TG: 19/26 CG: 18/23	A	10–14	3w 3 sessions	IPL only	Sham	3m
Xue [35]	New-Zealand	2020	87	TG_1_: 48 ± 15 TG_2_: 56 ± 17 CG: 55 ± 14	TG_1_: 9/19 TG_2_: 11/18 CG: 9/21	B	9–13	D0-D15-D45-D75 4 sessions	IPL only 1: 4f 2: 5f	Sham	15w
Yan [31]	China	2021	120	TG: 42.4 ± 14.2 CG: 41.8 ± 14.1	TG: 16/44 CG: 12/48	A	12–15	3w 3 sessions	IPL + MGX	WC + MGX	9w
Yin [34]	China	2018	35	TG: 41.6 ± 9.7 CG: 40.8 ± 14.0	TG: 9/9 CG: 9/8	A	16–17	1m 3 sessions	IPL only	WC	3m
Zarei-Ghanavati [38]	Iran	2021	100	TG: 44 ± 16 CG: 45 ± 16	TG: 18/32 CG: 13/37	B	11–13	D0-D15-D45 3 sessions	IPL only	WC	75d

Abbreviations: TG = Treatment group; CG = Control group; M/F = Male/Female; A = M22, Lumenis, Israel; B = E>Eye, E-SWIN, France; d = days, w = weeks; m = months; f = flashes; IPL = Intense Pulsed Light; MGX = Meibomian Gland Expression; WC = Warm Compresses and other eyelid hygiene measures; AB = antibiotics (tobramycine/dexamethasone).

## Data Availability

Data sharing is not applicable to this article as no new data was created.

## Supplementary Materials

The following supporting information can be downloaded at: https://www.mdpi.com/article/10.3390/jcm12083039/s1, Table S1: Data collected.

## Abbreviations

DED	Dry eye disease
IPL	Intense pulsed light
MGD	Meibomian gland dysfunction
MGX	Meibomian gland expression
NIBUT	Non-invasive break-up time
OSDI	Ocular surface disease index
SPEED	Standard patient evaluation of eye dryness
TBUT	Tear break-up time
