# Supplementary material for: Intense Pulsed Light Therapy in the Treatment of Dry Eye Diseases: A Systematic Review and Meta-Analysis
Source: J Clin Med. 2023 Apr 21;12(8):3039. doi: 10.3390/jcm12083039 (PMC10145895; doi:10.3390/jcm12083039)
Supplement: Supplementary file 1 [file jcm-12-03039-s001.zip › jcm-2305222-supplementary.pdf]

Supplementary Table S1. Data collected.

Data Collected and Used in Analyses	Data Collected and Used for Subgroups	Data Collected and Not Analyzed
- Sample size<br>- NIBUT<br>- TBUT<br>- OSDI	- Device characteristics<br>- Control group intervention<br>- Age<br>- Sex ratio	- Country of origin of the study<br>- Year of publication<br>- Treatment schedule and frequency<br>- Longest follow-up
